# Characterization of Chemosensory Responses on the Labellum of the Malaria Vector Mosquito, *Anopheles coluzzii*

**DOI:** 10.1038/s41598-018-23987-y

**Published:** 2018-04-04

**Authors:** Ahmed M. Saveer, R. Jason Pitts, Stephen T. Ferguson, Laurence J. Zwiebel

**Affiliations:** 1 Department of Biological Sciences, Vanderbilt University, Nashville, TN 37235 USA; 2 Present Address: Department of Biology, Baylor University, Waco, TX 76706 USA

## Abstract

*Anopheles gambiae coluzzii (An. coluzzii)* uses olfaction to modulate a range of critical behaviors that are essential for survival and reproduction; most notably, host preference and selection underlie its vectorial capacity for human malaria. As is the case for all mosquitoes, *An. coluzzii* has three specialized peripheral olfactory appendages—the antennae, maxillary palps and labella—which are used to detect and orient in response to a large variety of olfactory cues. Of these, neither the molecular nor the physiological significance of the labellum have been thoroughly characterized despite suggestions that labial-derived odorant reception is critical for close-range host attraction. Here we report global chemoreceptor transcriptome profiles together with a systematic electrophysiological analysis of labial T2 sensilla, and associated behavioral responses of female *An. coluzzii*. Single sensillum recordings of the T2 sensilla revealed robust responses to odorants previously associated with human sweat and oviposition sites and identified a 10-component blend that elicited attraction in a dual-choice landing bioassay designed to mimic host seeking in which non-blood fed females were significantly more attracted to the labial-responsive odorant blend as compared to gravid females. Taken together, these data suggest that, in *An. coluzzii*, olfactory responses derived from the labellum contribute to host-seeking.

## Introduction

Mosquito-borne diseases continue to have a major impact on global public health with almost half of the world’s population at risk for infection from malaria and arboviral pathogens. The incidence of malaria among high-risk populations has fallen over the last decade as a result of a range of malaria intervention programs^1^, but without new interventions, infections and deaths may surge once again. One promising approach is to exploit the mosquito’s reliance on olfaction for locating food sources, blood meal hosts and suitable oviposition sites^2,3^, to develop odorant-based methods to reduce disease transmission by preventing mosquito-human interactions^4,5^. *An. coluzzii* and *An. gambiae* (previously known as the ‘M’ and ‘S’ forms of the *An. gambiae* species complex^6^) are two closely related incipient species and the principal vectors of *Plasmodium falciparum*, the pathogen that is responsible for human malaria in sub-Saharan Africa. As is true for many, but not all, mosquitoes, blood meal host-seeking is an obligatory component of the life cycles of both *An. coluzzii* and *An. gambiae* and is integral in disease transmission^7^.

The mosquito olfactory apparatus is comprised of three specialized head appendages–the antennae, maxillary palp and labella—which are covered by specialized sensory hairs called sensilla that typically house two to three olfactory sensory neurons (OSNs). At the molecular level, OSNs are thought to express unique types of chemosensory receptors on dendritic membrane to detect a wide array of environmental odorants, reviewed in^8^. Similar to other insects, the genome of *An. gambiae* encodes three classes of chemosensory receptors^8^. These chemoreceptors include odorant receptors (*Ors*), ionotropic receptors (*Irs*) and gustatory receptors (*Grs*) that are known to detect a range of volatile odorant classes associated with mosquito host-seeking, oviposition and other several key behaviors that enhance their reproductive fitness^9–11^.

This study focuses on peripheral olfaction specifically localized in the labellum of the female proboscis, which remains the least characterized chemosensory appendage, but one that nevertheless appears to be extremely sophisticated^12–14^. Earlier studies on the labellum provided the first insights that this organ carries odor-sensitive neurons in T2 sensilla that evoked robust neuronal responses to a panel of odorants^13^. This work was interesting but did not encompass gene expression profiling or a more thorough characterization of T2 sensitivities and their behavior relevance. We hypothesize that the chemosensory responses derived from the labella mediate a distinct subset of close-range behavioral processes that are associated in the final stages of host-seeking and perhaps other essential behaviors such as oviposition. To examine this question, we initially performed RNAseq based transcriptome profiling of the male and female *An. coluzzii* labellum. We next performed a series of single sensillum electrophysiological recordings (SSRs) to characterize the T2 olfactory sensilla across the ventral surface of the labial lobe against a panel of volatile odorants. Finally, we also examined the behavioral saliency of labellar OSN activating odorants in laboratory based dual-choice landing and oviposition assays.

Our RNAseq based comprehensive transcriptome analysis of the proboscis labella and systematic electrophysiological assessment of T2 olfactory sensilla underscored that the mosquito proboscis labella are capable of detecting and discriminating a wide range of volatile odorants both individually and within the context of blends. Remarkably, although mosquito labella consist of a single morphological subtype of T2 sensilla, the tuning breadths of individual sensilla varied markedly along the labellum according to a spatially segregated cline. These electrophysiological studies identified a labial-active 10-component odorant blend that evoked robust behavioral responses in host-seeking females while gravid females seeking oviposition sites were indifferent to these blends. Taken together, these studies support the hypothesis that host-seeking female *Anopheles* mosquitoes detect olfactory cues from the proboscis labella that are likely to play a significant role in host selection and location behaviors that are integral to blood feeding.

## Results

### Chemosensory Transcripts in An. coluzzii Labella

**Figure 1:**
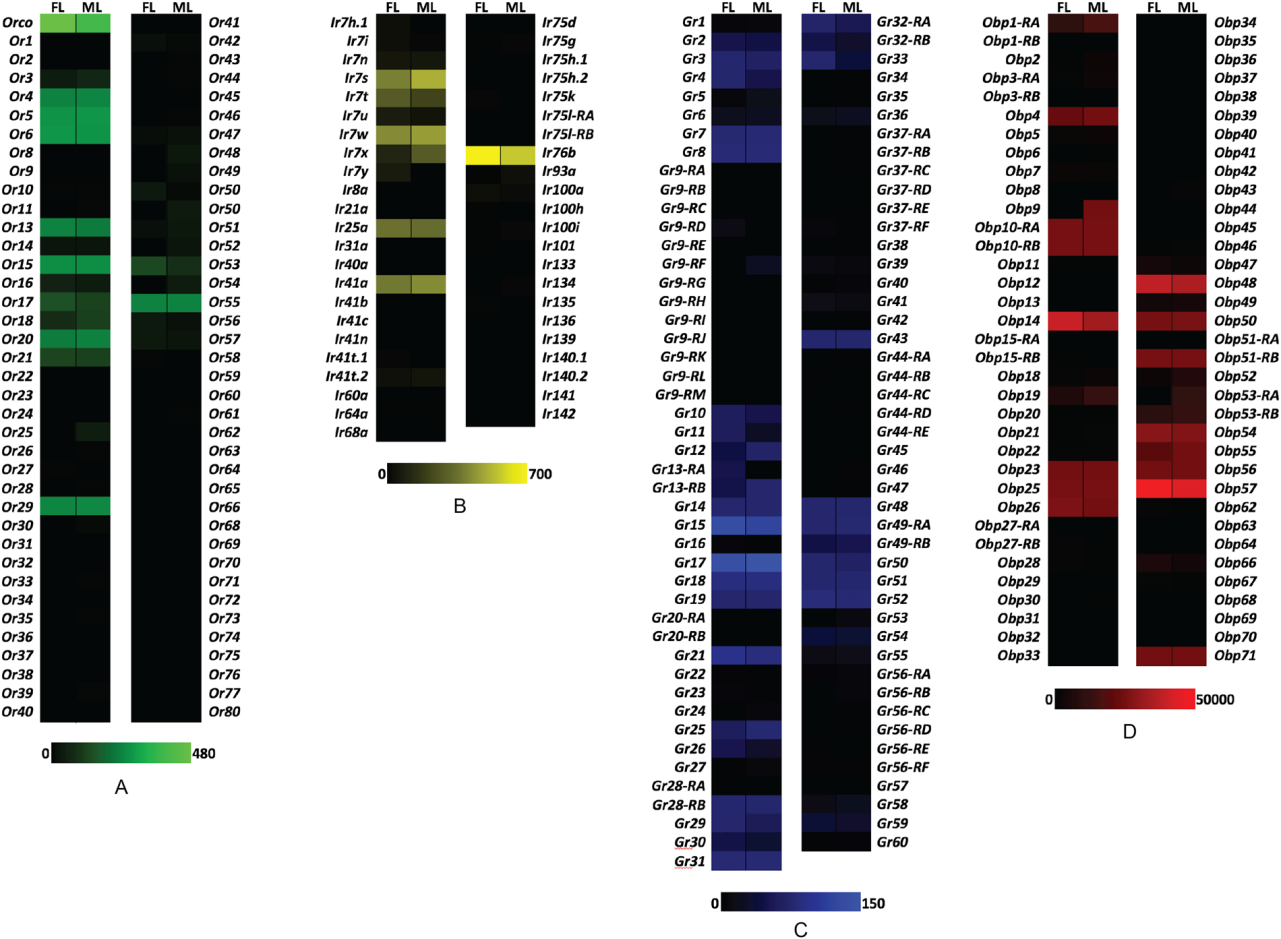
Chemosensory gene family expression profiles in *An. coluzzii* labella. (**A**) abundances of *AgOrs* (green). (**B**) abundances of *AgIrs* (yellow). (**C**) abundances of *AgGrs* (blue). (**D**) abundances of *AgObps* (red). Color intensity scales indicate increasing FPKM values from left to right. FL – female labella; ML – male labella.

To examine labellar transcript expression patterns, we carried out RNA sequencing from replicate samples of both female and male *An. coluzzii* adults. We were especially interested in the relative abundances of large multigene families of chemoreceptors, including the odorant receptors (*AgOrs*), variant ionotropic receptors (*AgIrs*), gustatory receptors (*AgGrs*) and odorant binding proteins (*AgObps*). Interestingly, the patterns of labellar-expressed transcripts within each of these gene families were very similar in both sexes (Fig. 1; supplementary Table S1), indicating a lack of sexual dimorphism in the labellar lobes of *An. coluzzii*. The transcriptome profile in the labellum (Fig. 1) revealed *AgOrco* as the most abundant odorant receptor, followed by a subset of ligand-specifying receptors including *AgOrs 3, 4, 5, 6* and 21 that are either absent or expressed at very low abundances in other adult chemosensory appendages^15^. This pattern corresponds well with previous characterizations of the *An. coluzzii* labellum^13^, where a small subset of *AgOrs* were shown to be functionally expressed. Similarly, a small subset of *AgIrs*, including the coreceptors, *AgIr25a* and *AgIr76b*, were expressed above a threshold of 3 FPKM in the labellum, suggesting that this family of chemoreceptors may be involved in chemical sensing in labellar sensilla (Fig. 1). We also observed the expression of a large number of *AgGr* transcripts (Fig. 1C) that are likely to function as contact chemosensors in the labellum and are mostly absent from adult head appendages^15^. Among these are *AgGrs* 14–21, homologs of the *Aedes aegypti* candidate sugar receptors^16^ that are also expressed in the labella of both sexes^17^. Also expressed in the labellum of both sexes of *An. coluzzii* is *AgGr25*, a receptor that is activated by monosaccharide and disaccharide sugars^18^. In light of the role that OBPs are thought to play in facilitating odor passage through the aqueous lymph of sensilla^8^, the extremely high expression levels of some members of the *AgObp* gene family (Fig. 1D) further supports the prediction that OSNs on the labella of *An. coluzzii* are sensitive to volatile odors.

### Single sensillum electrophysiology of labial OSNs

**Figure 2:**
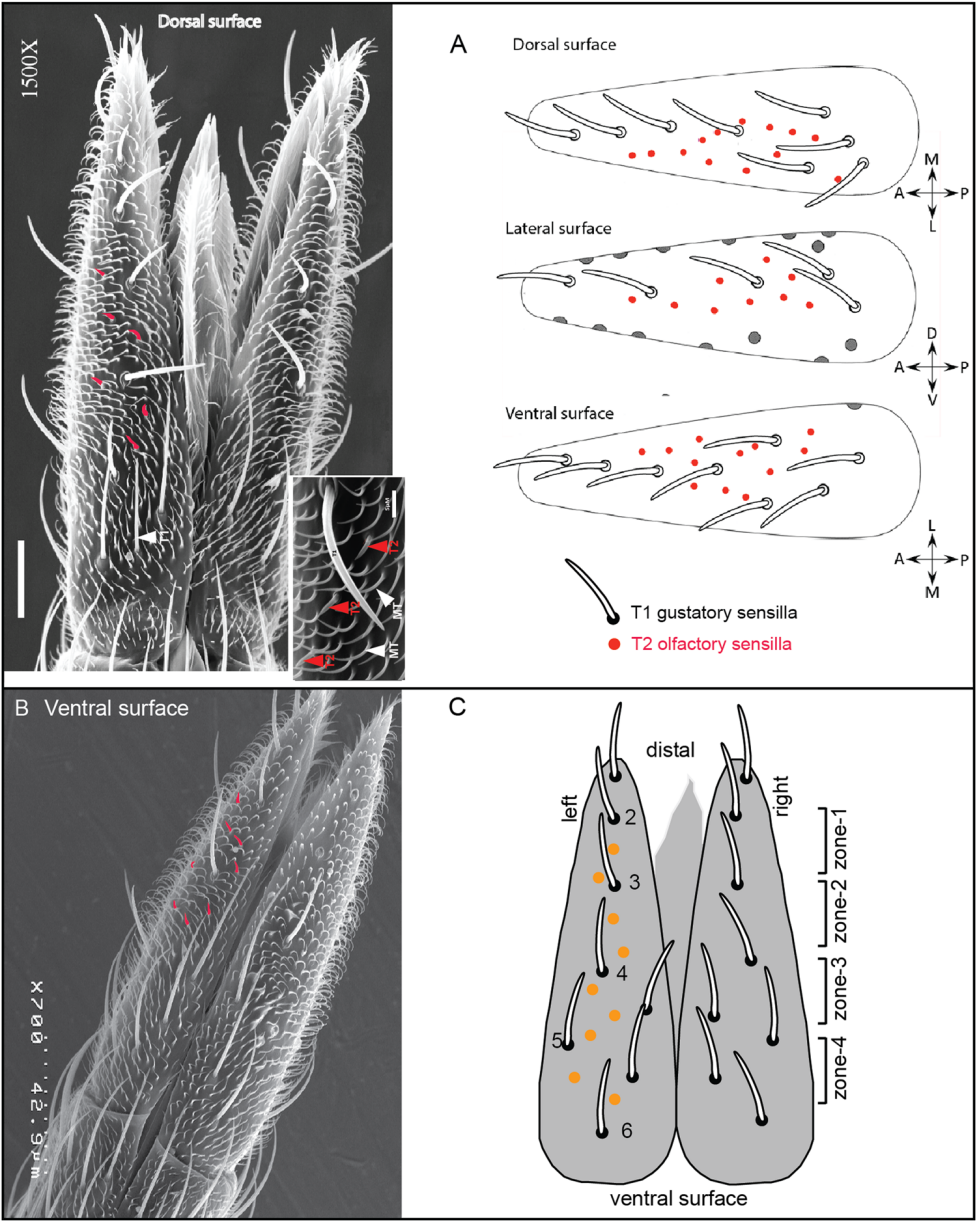
(**A**,**B**) Scanning electron micrograph and schematic of the dorsal and ventral side of the female *An. coluzzii* proboscis labellum. (**C**) Schematic ventral view of the *An. coluzzii* left and right labial lobes. Labellum is divided into 4 zones (Zone-1, 2, 3 and 4) based on the T1 gustatory sensilla location. Orange solid circles represent the SSR characterized T2 olfactory sensilla.

Given our previous characterizations of *Or* expression in the labella of *An. coluzzii*^12,13^, we sought to further define the localization of the T2 sensilla that potentially house OSNs by scanning electron microscopy. As shown in Fig. 2, the dorsal, ventral and lateral surfaces of the labellar lobes house a total of approximately 30 short, thorn-like T2 sensilla (inset) among a multitude of non-innervated microtrichia. The T2 sensilla reside in highly regular positions between the much longer T1 gustatory hairs on the external surface (Fig. 2A). The gustatory hairs could be used as landmarks to demarcate zones 1–4 from which to precisely perform electrophysiological recordings from T2 sensilla across different individuals (Fig. 2C).

**Figure 3:**
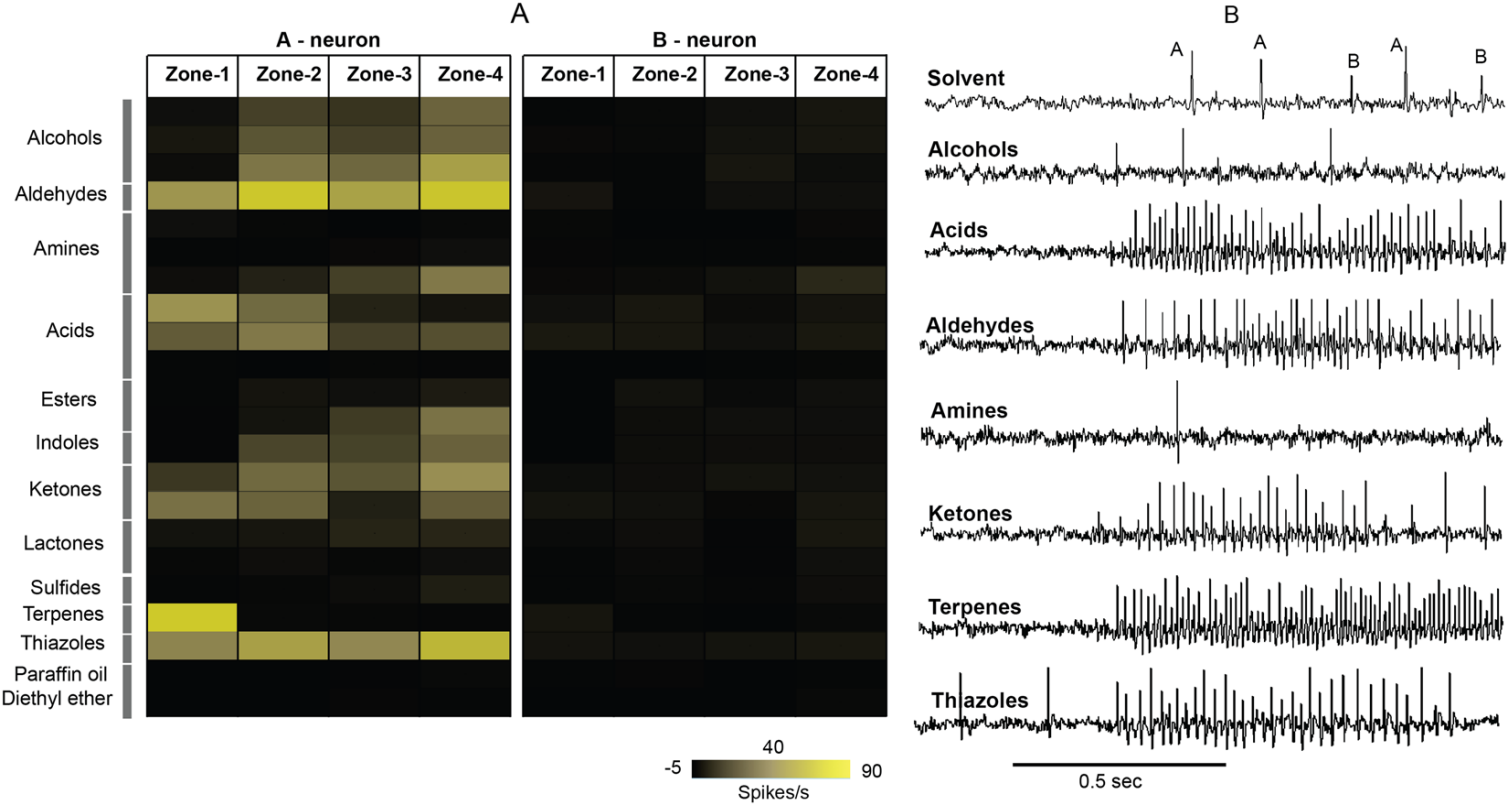
Odor coding in the labella of female *An. coluzzii* mosquitoes. (**A**) A heat map indicating the mean responses (spikes/s, SE, N = 2–3) of T2 olfactory sensilla across the four zones (Zone-1, 2,3 and 4) to a panel of 20 odorant blends belongs to 11 chemical classes. (**B**) Representative SSR traces of T2 olfactory sensillum located in the zone-1 to a panel of odor blends at 10^−2^M concentration (Solvent, alcohols, acids, aldehydes, amines, ketones, terpenes, thiazoles). The traces are temporally expanded to better illustrate the distinctive A- and B-neuron spike amplitudes in the solvent-alone control traces (top).

**Figure 4:**
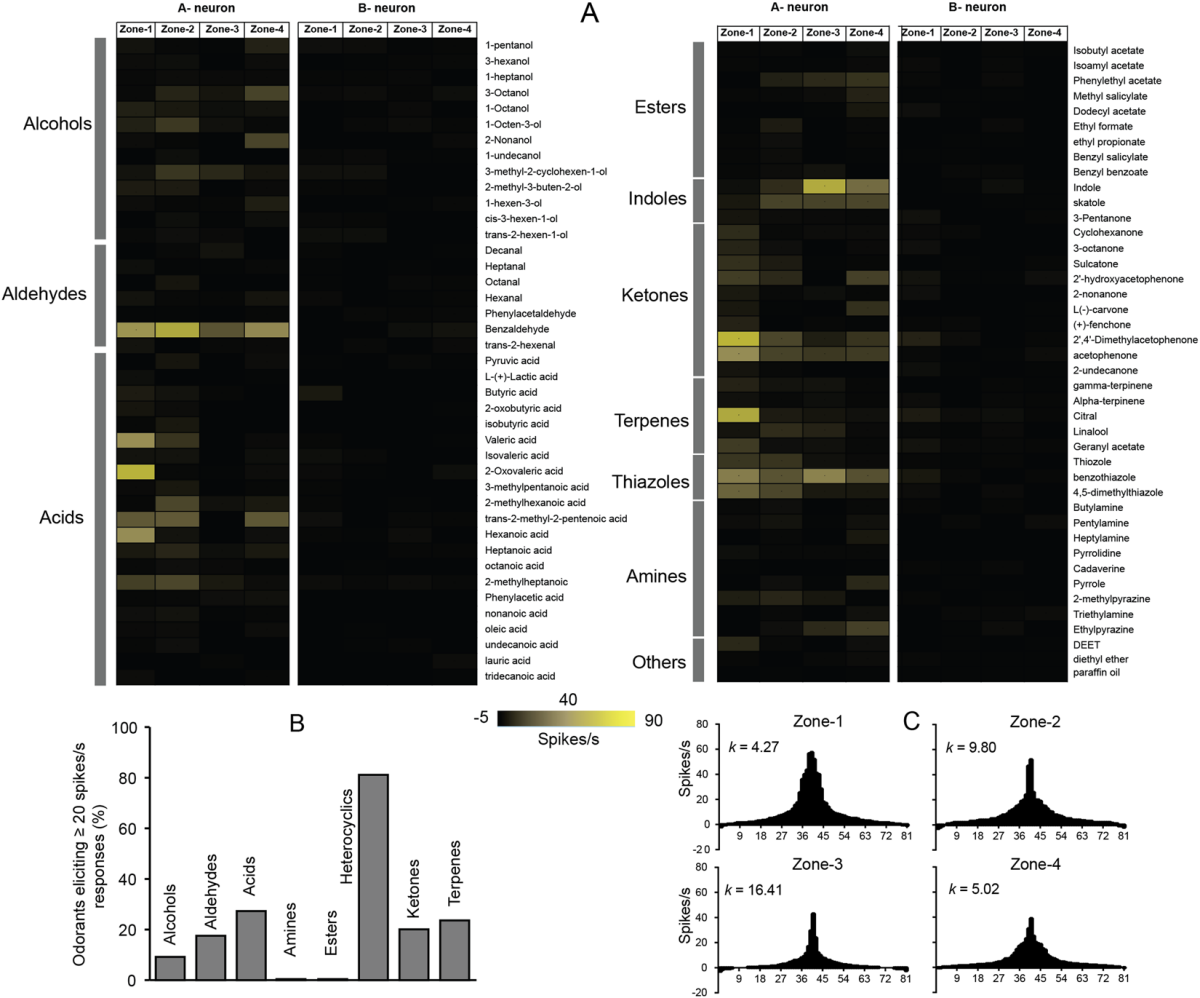
(**A**) A heat map indicating the mean responses (spikes/s) of T2 olfactory sensilla across the four zones (Zone-1, 2,3 and 4) to a panel of 81 odors (10^−2^M). (**B**) Percentage of odorants of various classes that evoked ≥20 spikes/s responses. (**C**) Tuning curves of T2 olfactory sensilla located in the four zones (Zone-1, 2, 3 and 4) analyzed by measuring the kurtosis (*k*) values (N = 2).

To better understand the nature of the olfactory cues that elicit responses from the *An. coluzzii* labella, we first examined the T2 olfactory sensilla using SSR against 20 customized blends of 94 unitary odorants spanning 11 chemically diverse classes, several of which are components of human odor and oviposition site volatiles^10,13,19,20^ (supplementary Table S2). Consistent with our initial study^13^, labial T2 olfactory sensilla house 2 OSNs giving rise to distinct spike amplitudes and markedly characteristic neuronal responses against our diverse panel of odorant stimuli (Fig. 3A). To begin with, the larger spike amplitude labial OSN (designated as the “A-neuron”) was significantly more sensitive to odor blends than the smaller spike amplitude “B-neuron” (Fig. 3C) which, in these assays, was largely unresponsive with the exception of T2 sensillum showing very weak responses (13 spikes/s) to an amine blend in zone-4 (Figs 3 and 4). Furthermore, with regard to the A-neuron response profiles, sensitivity to distinctive chemical class blends differed along the labellum from distal (zone-1) to proximal (zone-4) (Fig. 2C); with more response similarity within each zone than between the zones (Fig. 3A). The zone-1 T2 sensilla (N = 2–3) evoked very strong excitatory responses to blends made up of aldehydes (>40 spikes/s), carboxylic acids (>40 spikes/s), terpenes (>60 spikes/s) and thiazoles (>35 spikes/s). Interestingly blends made up of alcohols, amines, esters and indoles did not evoke any excitatory responses in zone-1 but evoked strong responses in the other zones (Fig. 3A and supplementary Table S3). Odorant classes such as aldehydes, acids, ketones and thiazoles evoked strong excitatory responses in the T2 sensilla across all the zones. Conversely, none of the T2 sensilla were stimulated by lactones and sulfur blends (Fig. 3A and supplementary Table S3).

In order to identify active unitary compounds from their respective blends, we excluded odorant classes that did not evoke significant excitatory responses (e.g. lactones and sulfides). For consistency, in light of the left and right bilateral symmetry with respect to sensilla positions and numbers exhibited between labial lobes (Fig. 2), we focused our SSR studies on T2 sensilla dispersed along the left labellar lobe. As was observed for responses to stimuli blends, none of the unitary compounds tested evoked noticeable responses in the labial B-neurons (Fig. 4A and supplementary Table S5). Furthermore, of the 81 unitary odorants, only 19% evoked robust (≥20spikes/s) excitatory responses across all the zones (Fig. 4A and supplementary Table S4). These consisted of mostly aldehydes, acids, heterocyclics, ketones and terpenes (Fig. 4B). In particular, among the aldehydes and terpenes, benzaldehyde and citral (a known plant-based insect repellent used widely against mosquitoes^21^) evoked strong (>50 spikes/s) excitatory responses that largely accounted for the overall response of the entire blend. Of the 21 carboxylic acids tested, only five (23%)—valeric acid, 2-oxovaleric acid, trans-2-methyl-2-pentanoic acid, hexanoic acid and 2-methylheptanoic acid—evoked neural responses (≥20 spikes/s). Some of these compounds are known to be components of human odor and important host cues for the mosquito *An. gambiae*^22–25^.

With few exceptions, we observed generally consistent neuronal responses between blends and unitary compounds within chemical classes. The exceptions were alcohols, amines and esters that evoked very weak responses when delivered singly (Fig. 4A). In order to visualize the tuning breadths of the T2 sensilla across the labella, odor-tuning curves were generated for each zone and their kurtosis values (k values) were measured. The tuning curves of the four zones to the 81 odorants across the labella revealed a bimodal distribution (Fig. 4C) with broadly tuned T2 sensilla localized in zones-1, 2 and 4 (k = 4.27, 9.8, and 5.02) and narrowly tuned sensilla in zone-3 (k = 16.41).

### Host-seeking mosquitoes are attracted to labial active blend

To test whether odorants that are electrophysiologically active against labial T2 sensilla of *An. coluzzii* are behaviorally effective, we employed laboratory bioassays that examine olfactory inputs for two critical behaviors for adult female mosquitoes: host seeking and oviposition. For these studies, we employed an odorant blend selected on the criteria of eliciting the most robust excitatory responses (≥25 spikes/s) in our labial T2 sensillar SSR analyses. In this manner, we selected 11 compounds; of these, we excluded citral because of its previously reported role as a mosquito repellent^21^. Our final blend therefore consisted of 10 compounds (benzaldehyde, trans-2-methyl-2-pentanoic acid, hexanoic acid, valeric acid, 2-oxovaleric acid, indole, acetophenone, 2,4-dimethylacetophenone, 4,5-dimethylthazole and benzothiazole; supplementary Table S5) that evoked robust neuronal responses across the labella.

**Figure 5:**
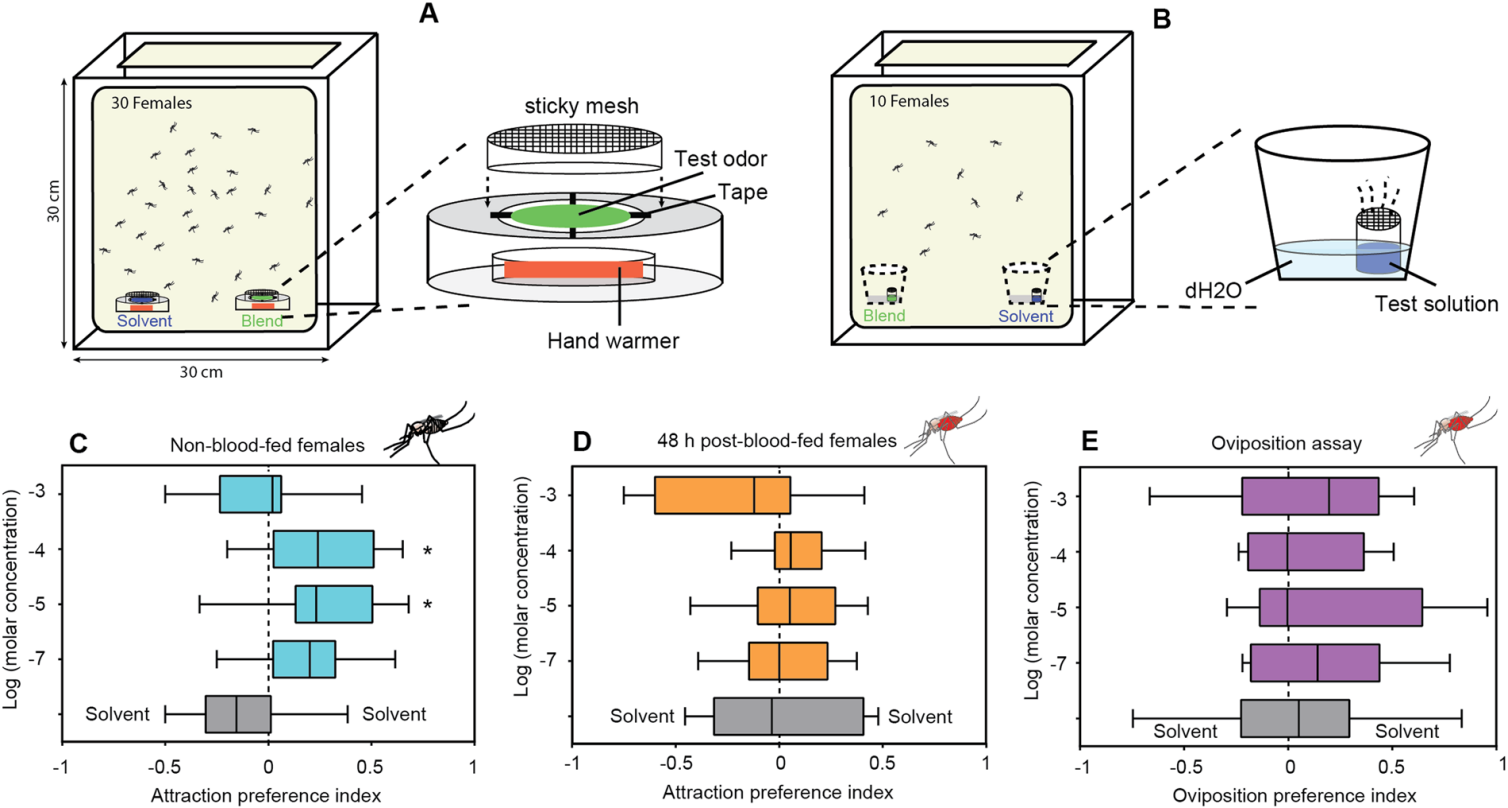
Behavioral responses of the *An. coluzzii* to labella-active odorant blend. Schematic representation of the dual-choice heat based landing assay (**A**) and oviposition (**B**) assays. Landing preference of non-blood-fed (**C**), and 48 h post-blood-fed (**D**) female mosquitoes to a 10-component labial active blend (mean ± s.e.m.; N = 10). (**E**) Oviposition preference of gravid mosquitoes to a labial active blend (mean ± s.e.m.; N = 8). The landing and oviposition index for the four doses of the blend were compared to ethanol control (*P < 0.05, one-way ANOVA with Bonferroni’s post-test).

This blend of SSR-active compounds was initially tested in a dual-choice landing assay designed to mimic host preference against non-blood-fed (host-seeking) and post-blood-fed (gravid) female *An. colluzzii* mosquitoes (Fig. 5A). In these studies, non-blood-females are significantly attracted towards the 10-component blend in a dose dependent manner (Fig. 5C), and this attraction is notably abolished after blood feeding (Fig. 5D). Interestingly, of the four concentrations tested, significantly more non-blood-fed females were attracted to the two intermediate doses and showed strong tendency at the lowest dose when compared against an ethanol control (Fig. 5C). However, at the highest concentration, females were either indifferent or exhibited slight avoidance behavior (Fig. 5C). This attraction is abolished as a result of blood feeding, i.e. none of the 48 h post-blood-fed females were significantly attracted to any of the four concentrations of the blend when compared with a solvent (Fig. 5D). We next tested the behavioral responses of gravid mosquitoes against the four concentrations of labial blend in a dual-choice oviposition assay (Fig. 5B). Of the four tested concentrations, gravid mosquitoes did not show any preference to any of the tested doses (Fig. 5E).

## Discussion

We have previously shown the expression of a highly-conserved odorant co-receptor (*AgOrco*) throughout the female *An. coluzzii* labella^12^ as well as robust olfactory responses of single labial T2 sensillae against a limited odorant panel (~20 odors)^13^. The current study uses RNAseq to carry out a comprehensive analysis of the chemosensory transcriptome profiles on the labellum as well as a significantly broader characterization of the neural responses across the ventral T2 olfactory sensilla against 94 compounds of various chemical classes than had been carried out previously^12^. Finally, we performed a series of direct behavioral validations of those responses against a subset of labellar-active compounds.

To begin with, we characterized the transcript abundance profiles of the labellar lobes of females and males using RNAseq to reveal highly similar expression patterns for the three major chemoreceptor gene families as well as odorant binding proteins between sexes (Fig. 1). However, the transcript abundance patterns of these gene families are remarkably distinct from those previously shown for antennae and maxillary palps in this species^15^. Of particular note are the transcripts for *AgOrs 3, 4, 5, & 21* and *AgIrs 7n, 7s, 7*x*, & 7y* that seem to be specifically expressed in the labellum, suggesting a specialized function for detection of volatile odors by these chemoreceptors in this appendage. The presence of *AgIr25a* and *AgIr76b* co-receptors that are required for functional activation of amine sensitivity^26,27^, suggests that *AgIrs* in the labellum may be responsible for the observed responses to amine blends in zone 4 (Fig. 3B; Supplementary Table S3), and compounds like pyrrole, 2-methylpyrazine, and ethylpyrazine (Fig. 4A; Supplementary Table S4).

In addition to the presence of a significant subset of the chemosensory receptor and OBP repertoire of *An. coluzzii* which underscores the complexity and significance of the labial olfactory response, our studies revealed that labial T2 OSNs can detect and discriminate a wide range of volatiles. Indeed, robust neural responses to odorant classes such as aldehydes, acids, ketones, heterocyclics and terpenes that are associated with human sweat and oviposition site odors were detected. As expected, we observed two categories of OSNs based on their response profile: narrowly and broadly tuned T2 olfactory sensilla. Interestingly, the observed neural response profiles were distinct and spatially segregated from the distal to proximal ends of the labellum. These variable response profiles may either be due to our odorant panel lacking the principal cognate ligand of certain receptors or, perhaps more likely, different classes of olfactory receptors are expressed in the T2 sensilla that are spatially segregated across the labella. The later explanation is more evident from our recordings as citral evoked the strongest neural response only in zone-1 T2 sensilla. Notably, citral is a plant-based insect repellent used as a personal protection against host-seeking mosquitoes^21^. Among other odorant classes tested, carboxylic acids such as oxovaleric acid, pentanoic acid, hexanoic acid and heptanoic acid, which are known to be a part of human sweat and are important cues for host-seeking mosquitoes^24,25,28,29^, evoked robust neural responses and are likely to be sensed by the *AgIrs*^27^.

The fact these neuronal response profiles are spatially segregated across the labial lobes suggests that odorant sensitivities may also reflect proximity to odor sources over very short distances^30^. This, in turn, may mediate the actual selection of blood-feeding site during the penultimate stages of the host-seeking behavior. Indeed, intra-vital video microscopy of feeding behavior in *An. gambiae* has revealed prolonged probing of skin by the proboscis both before and after penetration^31^. Similarly, Orco expressing olfactory neurons were recently discovered on the tip of the *Manduca sexta* proboscis and found to be involved in evaluating the quality of individual flowers during pollination^32^.

In an effort to test the behavioral relevance of labial odorant responses, we have used a landing-based bioassay to show that blood-meal host seeking female *An. coluzzii* display robust and dose-dependent attraction to a blend of labellar-active volatiles. We initially selected 11-compounds that evoked robust SSR responses (≥25 spikes/sec). However, we excluded citral from the blend and measured mosquito host-seeking and oviposition behaviors against a 10-component blend (supplementary Table S5) in laboratory-based dual choice landing and oviposition assays. Host-seeking female *An. coluzzii* mosquitoes were attracted towards the labial active blend under laboratory conditions, and the responses were dose-dependent and subjected to physiological modulation as gravid females (48 h post blood-fed) were indifferent to the blend in landing as well as oviposition assay. The lack of responses observed in gravid females provides compelling evidence to suggest they are narrowly specific to the highly-specialized task of mosquito host-seeking and the reproductively imperative acquisition of blood-meals.

Taken together, these results suggest that the odorants that activate labial T2 olfactory sensilla plays an important role in host-seeking rather than oviposition behavior. However, as these volatile odorants are also known to activate *An. coluzzii* antennal olfactory sensilla^33^ additional experiments are required to elucidate the precise role of the labellum in mosquito host-seeking behaviors. It is noteworthy however that the OR-expressing neurons in the proboscis of *An. gambiae* project both to the primary olfactory center – the antennal lobe (AL)^13^ and the primary taste center, the subesophageal zone (SEZ)^14^, suggesting a sensory integration of olfactory and gustatory stimuli, an intriguing question that remains to be explored. An enhanced understanding of the molecular basis of odor coding, including the topographic distribution of *Or*s and functional characterization of all labial-specific olfactory receptors (*AgOr*s and *AgIrs*) would augment the identification of compounds that either stimulate or inhibit OSNs in mosquitoes, thus facilitating the development of effective close range mosquito repellents^4,9,34^.

## Materials and Methods

### Mosquito rearing and preparation

*Anopheles coluzzii* (SUA 2La/2La), previously known as *Anopheles gambiae* s.s. M form^6^ and originating from Suakoko, Liberia, was reared and maintained in a walk-in climate chamber at 27 °C, 75% relative humidity (RH) under a 12:12 L:D photoperiod as described previously^10^. In brief, mosquito eggs were collected in an egg cup filled with dH_2_O; post-eclosion, the larvae were reared in trays (~400 per tray) filled with 1 L dH_2_O. Bioassays were conducted with ~200 non-blood-fed (nBF) and 48 h post-blood-fed (pBF) females of 5- to 8-days old from the same cohort and transferred to a separate cage with constant access to 10% sucrose solution. For blood feeding, 5- to 6- days old females were provided sheep blood (Hemostat Laboratories Inc. Dixon, CA) by using an artificial membrane feeding system (Hemotek, Lancaster, UK).

### Scanning Electron Microscopy

Scanning Electron Microscopy (SEM) imaging was performed as described previously^35^ (detailed methods are provided in the supplemental data).

### RNA sequencing and analysis

Four-to-six day-old adult female and male *An. coluzzii* mosquitoes were collected in the middle of the light phase (~ZT6) and chilled at 4 °C. For each collection, labellar lobes were hand-dissected into TRIzol and total RNA was isolated. mRNA isolation and cDNA library preparation were carried out using the Illumina mRNA sequencing kit (Illumina Inc.; San Diego, CA). Libraries were barcoded and sequenced in single-end fashion (50SE) on an Illumina HiSeq. 2000. Two independent, biological samples were collected for each sex. Individual Illumina read files (FASTQ) were trimmed prior to mapping to the *An. gambiae* reference genome (version AgamP4) using Tophat2 (version 2.0.8) with the guidance of gene annotation (version AgamP4.4). Weighted alignments were reported for each mapped read. Fragments per kilobase per Million (FPKM) was calculated using Cufflinks software^36^.

### Single Sensillum Recordings

SSRs were conducted on 5–8 days old non-blood-fed females using the same experimental protocols described previously^10^. Briefly, a female mosquito was cold immobilized (~1 min at – 20 °C) and mounted on a microscope glass slide (25 × 75 × 1.0 mm) using double-sided sticky tape. The head appendages, including the labellum, were mounted on double-sided tape with the labrum removed using a fine paintbrush and oriented to allow optimal access to the T2 sensilla. SSRs were performed on the left labial lobe on the ventral surface of the labellum under a microscope (BX51WI Olympus) viewed at 1200 × magnification. Two glass capillaries with filament (OD-1.5 mm, ID-0.84 mm, WPI, Inc. Sarasota, FL) inserted with chloridized silver wire (0.25 mm diameter, Sigma-Aldrich) and filled with 0.1 M KCl saline were used as a reference and recording electrode, respectively. The reference electrode was placed in the eye, and the recording electrode was attached to a preamplifier (Syntech universal AC/DC 10×, Syntech, Hilversum, The Netherlands) whereby its tip was inserted into the base of a T2 sensillum to establish electrical contact with the OSNs using a piezo micromanipulator (PPM5000 WPI, Sarasota, FL). The signals were digitized by the IDAC4 interface box (Syntech, Hilversum, The Netherlands), and the data were recorded and analyzed using Auto Spike v. 3.2 software (Syntech, Hilversum, The Netherlands).

A charcoal-filtered humidified air-flow (0.5 L min^−1^) was continuously directed at the preparation through a 19.5 cm long glass tube, which terminated ~15 mm from the labellum. Odor stimulation were carried out by blowing a 0.5 s air pulse (0.5 L min^−1^) controlled by a stimulus controller (Syntech, Hilversum, The Netherlands) through the stimulus pipette which was inserted into a hole in the glass tube.

The extracellular activity of OSNs in each T2 sensillum was characterized based on the differences in the spike amplitude, spike frequency and shape as described in our previous study^13^. OSNs with larger spike amplitudes were described as “A” cells and those with smaller spike amplitudes as “B” cells as described previously^13,37^. The change in neural activity was determined by manually counting number of spikes 1000 ms after the onset of neuronal response minus the number of spikes 1000 ms prior to stimulus. All the SSR recordings were conducted on the T2 sensilla situated on the ventral surface of the left labial lobe (Fig. 2A). Four zones (Zone 1, 2, 3 and 4) were made based on the location of the long T1 gustatory sensilla from the distal to proximal end of the labellum. Zone 1, 2, 3 and 4 are located between the gustatory sensillum 2–3, 3–4, 4–5 and 5–6, respectively (Fig. 2A).

### Chemical stimuli, preparation and stimulation

Electrophysiological screening of the T2 sensilla were done using 20 odorant blends of 11 different classes that consists of 94 unitary compounds at 10^−2^M concentrations (supplementary Table S1; more detailes provided in the supplementary data). We at first screened a total of nine T2 olfactory sensilla from the four zones with 2–3 T2 sensilla in each zone (N = 2–3) was tested against a panel of 20 odorant blends (supplementary Table S1). The blends that did not evoke any neuronal responses were excluded from the panel. We next examined the individual blend components (81 odors) at 10^−2^M concentration two times (N = 2) against each T2 sensillum across all the zones except for zone-2 where only one recording was performed on a single T2 sensillum (N = 1).

### Dual-choice landing bioassay

A dual-choice landing bioassay was used to access the behavioral responses of non-blood-fed (nB) and 48 h post-blood-fed (pBF) females to a range of labial active compounds that were delivered in the form of a blend. A pair of heat-based mosquito trapping systems were constructed (detailes provided in the supplemental data) for treatment (blend) and control (solvent) conditions using hand warmers, petri dishes, nylon mesh and sticky gel as described previously with modification^38^.

For each cage, two traps were placed side-by-side holding solvent and odorant treated filter discs, respectively. Location of solvent- and odorant-treated choice was rotated and the positions of the assay cages were randomly placed between each trial. Each day, 10 bioassay cages were prepared (5 non-blood-fed and 5 post-blood-fed females) that included control (solvent-solvent) and four concentrations (10^−3^, 10^−4^, 10^−5^ and 10^−7^M) of labial active blend (supplementary Table S5) for each physiological state. Odorant treated filter discs were prepared by impregnating the Whatman #1 filter paper disc (5.5 cm) with 200 µl of test odorants followed by solvent evaporation for ~10 min in the fume hood. Ethanol was used as a solvent control and to make decadic dilutions of labial active blend. Bioassay preparation was begun approximately 2 h prior to scotophase in the climate chamber under the same condition as described above by placing the two-choice trapping system with activated hand-warmers. For each replicate, 30 females were collected in a “release cage” (9.5 cm diameter × 10 cm long) closed with gauze and kept in the assay cage for 1 h before testing. Females were allowed to enter the cage by removing the gauze 15 min. before the scotophase (11:00). The total number of trapped female mosquitoes on the sticky gel, as well as non-responders, were counted the following day (09:00).

### Oviposition bioassay

The oviposition preference of gravid mosquitoes was tested in a dual-choice assay in a Bugdorm® insect rearing cage (polypropylene, 30 × 30 × 30 cm) as described previously with slight modification^10^. Briefly, two egg cups (PET, top opening = 7.0 cm, height = 6.0 cm, bottom = 5.0 cm) filled with 10 mL of dH2O as an oviposition substrate were placed in opposite corners of the assay cage. A pair of borosilicate glass vials (14.65 × 19 mm; Qorpak, Bridgeville, PA) with a screen top (10 × 10 mm) filled with 1 mL of test solution, labial active blend and solvent control were placed inside the egg cup (Fig. 5B). Experiments were started approximately 1 h prior to scotophase (11:00) by introducing 10 gravid females (48-hour pBF) into the assay cage through the ‘releasing chamber’ as described above, and the total number of eggs in the two ovipoistion cups were manually counted on the following day (09:00). Ethanol was used as a solvent control and to dissolve labial active blend. Ten bioassay cages were prepared each day with two replications for every treatment that includes control (solvent-solvent) and four concentrations (10^−3^, 10^−4^, 10^−5^ and 10^−7^ M) of labial active blend under the same climatic condition described above.

### Data analysis

The dual-choice attraction preference index (AI) and oviposition preference index (OI) were calculated using the formula AI (or) OI = (B − C)/(B + C); where ‘B’ is the total number of mosquitoes trapped on the sticky gel or eggs associated with the labial active blend and ‘C’ is the number of mosquitoes or eggs associated with the control odorants. The attraction and oviposition index were analyzed using a one-way ANOVA followed by Bonferroni’s multiple comparisons test to compare control (solvent) with all other doses of blend (GraphPad Prism; version 5.0a).

## Electronic supplementary material

Transcript abundances in An. coluzzii labella, expressed as Fragments per Kilobase per Million (FPKM).

Supplementary Information
