# Supplementary material for: Characterization of Chemosensory Responses on the Labellum of the Malaria Vector Mosquito, Anopheles coluzzii
Source: Sci Rep. 2018 Apr 4;8:5656. doi: 10.1038/s41598-018-23987-y (PMC5884837; doi:10.1038/s41598-018-23987-y)
Supplement: Supplementary file 2 — Supplementary Information [file 41598_2018_23987_MOESM2_ESM.pdf]

Supplementary Information for:

Characterization of Chemosensory Responses on the Labellum of the Malaria Vector Mosquito, *Anopheles coluzzii*

Author Affiliations:

Ahmed M. Saveer[1], R. Jason Pitts[1,2], Stephen T. Ferguson[1], Laurence J. Zwiebel[1a]

[1] Department of Biological Sciences, Vanderbilt University, Nashville, TN 37235, USA.

[2] Present Address: Department of Biology, Baylor University, Waco, TX 76706, USA.

[a] To whom correspondence should be addressed: l.zwiebel@vanderbilt.edu

Material and Methods

Scanning Electron Microscopy

Anopheles coluzzii female mosquito labella were fixed in 4% paraformaldehyde in phosphate-buffered saline (PBS) containing 0.1% Triton X-100, followed by dehydration using an ethanol series and the labella were dried in a fume hood. The samples were glued onto aluminum pin mounts with colloidal silver paint and sputter coated with thin film of gold-palladium. Samples were viewed using a Hitachi S-4200 SEM and were analyzed using Quart PCI version 6.0 image software (Quarts Imaging Corp. Vancouver, B.C.).

Chemical stimuli, preparation, and stimulation

We prepared blends based on the number of carbons except for amines which were grouped into primary, secondary and tertiary amines. Each blend consists of 3 – 8 components (supplementary table S1): three alcohol blends (Alcohol #1, 2,3); three amine blends (Amines #1,2,3); three carboxylic acid blends (Acids #1,2,3); two ester blends (Esters #1,2); two ketone blends (Ketone #1,2); two lactone blends (Lactone #1,2); as well as a single blend of aldehydes (Aldehyde #1); sulfurs (Sulfur #1), terpenes (Terpene #1) and thiazoles (Thiazole #1). All odorants were diluted in paraffin oil except for acids and amines which were dissolved in diethyl ether. Odor stimuli were applied as 25µL aliquots on a 10 × 30mm piece of Whatman # 1 filter paper that was inserted into a Pasteur pipette (14.5cm long, VWR International). Pipettes with paraffin oil and diethyl ether were used as controls. Each odorant pipette was not used more than three times. The loaded pipettes were kept open for 10-15 min in a fume hood to evaporate the solvent and then their wider ends were covered with 1 mL pipette tips.

Dual-choice landing bioassay

Air-activated hand warmers (HotHands® warmers, Heat Max, Dalton, GA) were used as heat sources (supplementary fig S1) and fitted into a 100 x 15mm petri dish base. These heat source petri dish bases were placed in the center of a larger 150 x 20mm petri dish base covered with a lid that had a 7 x 7cm window opening centered over the heat source situated underneath. Four pieces of 1.5 x 1.5cm double-sided tape were secured over the cut edges of the lid to hold the solvent and odorant treated filter discs in-between the heat source and sticky mesh. Another 100 x 15mm petri dish lid that had 7 x 7cm opening window was used as a frame to hold a nylon mesh disc, 9cm in diameter, coated with sticky gel (Tangle-Trap® sticky coating, ORTHO Group, Marysville, OH) and placed directly over the larger petri dish holding the solvent and odorant treated filter disc (Figure 5A). The perforated sticky mesh allowed odorants to pass through and at the same time trap attracted mosquitoes while not allowing mosquitoes to come in direct contact with the odor source (Figure 5A). In this manner, we ensured the observed behavior was mediated by olfactory (non-contact) chemosensory cues. The sticky mesh was used only once in each trial to avoid any contamination between the trails.

Supplementary figure S1. Temperature of HotHand hand warmers used as a heat source in the dual-choice landing assays was measured (n=5) overnight.

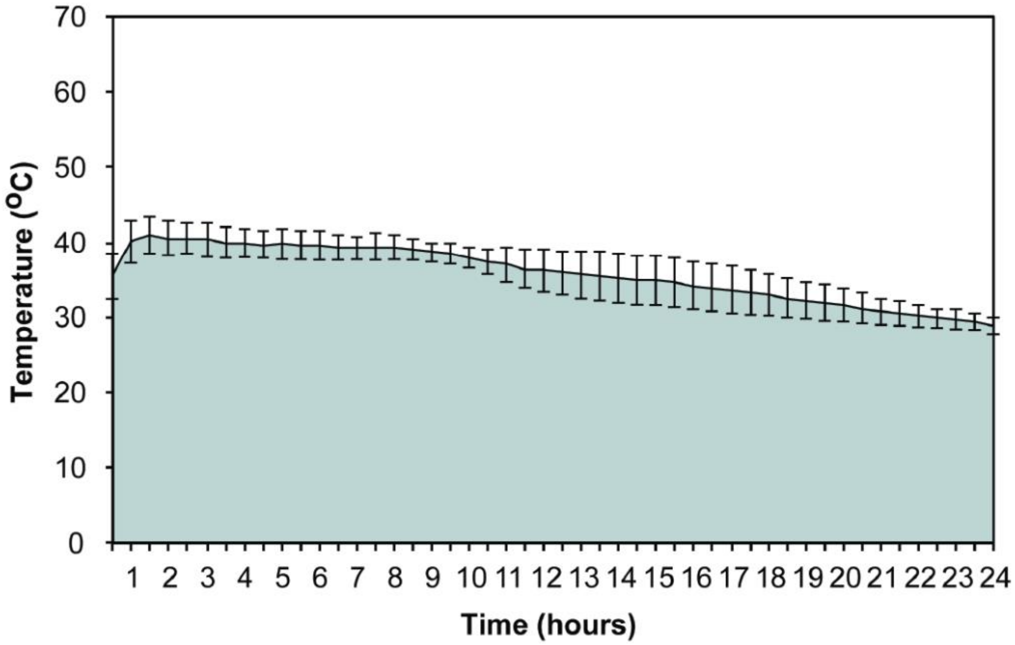

Supplementary table S1. Transcript abundances in *An. coluzzii* labella, expressed as Fragments per Kilobase per Million (FPKM).

Supplementary table S2. List volatile odorant compounds used in this study.

	Common name	IUPAC name	CAS#
Alcohol#1	1-pentanol	pentan-1-ol	71-41-0
	3-hexanol	hexan-3-ol	17015-11-1
	1-heptanol	heptan-1-ol	111-70-6
	3-octanol	octan-3-ol	5340-36-3
Alcohol#2	1-octanol	octan-1-ol	111-87-5
	1-octen-3-ol	oct-1-en-3-ol	3391-86-4
	2-nonanol	nonan-2-ol	628-99-9
	1-undecanol	undecan-1-ol	112-42-5
Alcohol#3	methyl-2-cyclohexenol	3-methyl-2-cyclohexen-1-ol	21378-21-2
	methylbutenol	2-methyl-3-buten-2-ol	115-18-4
	1-hexen-3-ol	hex-3-en-1-ol	4798-44-1
	1-heptene-3-ol	hept-1-en-3-ol	4938-52-7
	2-hexenol	trans-2-hexen-1-ol	928-95-0
Aldehydes	decanal	decanal	112-31-2
	heptanal	heptanal	111-71-7
	octanal	octanal	124-13-0
	hexanal	hexanal	66-25-1
	phenylacetaldehyde	2-phenylacetaldehyde	122-78-1
	benzaldehyde	benzaldehyde	100-52-7
	trans-2-hexenal	(E)-hex-2-enal	6728-26-3
Amines#1	butylamine	Butan-1-amine	109-73-9
	pentylamine	Pentan-1-amine	110-58-7
	heptylamine	heptan-1-amine	111-68-2
Amines#2	pyrrolidine	Pyrrolidine	123-75-1
	cadaverine	Pentane-1,5-diamine	462-94-2
	pyrrole	1*H*-Pyrrole	109-97-7
Amines#3	methylpyrazine	2-Methylpyrazine	109-08-0
	triethylamine	N,*N*-Diethylethanamine	121-44-8
	ethylpyrazine	2-ethylpyrazine	13925-00-3

Acids#1	pyruvic acid	2-oxopropanoic acid	127-17-3
	L-(+)-Lactic acid	2-hydroxypropanoic acid	79-33-4
	butyric acid	butanoic acid	107-92-6
	2-oxobutyric acid	2-oxobutanoic acid	600-18-0
	isobutyric acid	2-methylpropanoic acid	79-31-2
	valeric acid	pentanoic acid	109-52-4
	isovaleric acid	3-methylbutanoic acid	503-74-2
	2-oxovaleric acid	2-oxopentanoic acid	1821-02-9
Acids#2	DL-3-methylvaleric acid	3-methylpentanoic acid	105-43-1
	2-methylhexanoic acid	2-methylhexanoic acid	4536-23-6
	trans-2-methyl-2-pentenoic acid	(E)-2-methylpent-2-enoic acid	16957-70-3
	hexanoic acid	hexanoic acid	142-62-1
	heptanoic acid	heptanoic acid	111-14-8
	caprylic acid	octanoic acid	124-07-2
	2-methylheptanoic acid	2-methylheptanoic acid	1188-02-9
	phenylacetic acid	phenylacetic acid	103-82-2
Acids#3	nonanoic acid	nonanoic acid	112-05-0
	oleic acid	(9*Z*)-Octadec-9-enoic acid	112-80-1
	undecylic acid	undecanoic acid	112-37-8
	lauric acid	dodecanoic acid	143-07-7
	tridecylic acid	Tridecanoic acid	539-53-9
Esters#1	isobutyl acetate	2-methylpropyl ethanoate	110-19-0
	isoamyl acetate	3-methylbutyl acetate	123-92-2
	phenylethyl acetate	2-phenylethyl acetate	103-45-7
	methyl salicylate	methyl 2-hydroxybenzoate	119-36-8
Esters#2	dodecyl acetate	dodecyl acetate	112-66-3
	ethyl formate	ethyl formate	109-94-4
	ethyl propionate	ethyl propanoate	105-37-3
	benzyl salicylate	benzyl 2-hydroxybenzoate	118-58-1
	benzyl benzoate	benzyl benzoate	120-51-4
Indole	indole	indole	120-72-9
	3-methylindole/skatole	3-methyl-1*H*-indole	83-34-1

Ketones#1	3-pentanone	pentan-3-one	96-22-0
	cyclohexanone	cyclohexanone	108-94-1
	3-octanone	octan-3-one	106-68-3
	sulcatone	6-methyl-5hepten-2-one	110-93-0
	acetophenone	1-phenylethan-1-one	98-86-2
	2'-hydroxyacetophenone	1-(2-hydroxyphenyl)ethanone	118-93-4
Ketones#2	2-nonanone	nonan-2-one	821-55-6
	(+)-fenchone	1,3,3-Trimethylbicyclo[2.2.1]heptan-2-one	4695-62-9
	2',4'-dimethylacetophenone	1-(2,4-dimethylphenyl)ethanone	89-74-7
	2-undecanone	Undecan-2-one	112-12-9
Lactones#1	γ-Valerolactone	5-methyldihydrofuran-2(3H)-one	108-29-2
	γ-Hexalactone	5-ethyloxolan-2-one	695-06-7
	γ-Heptalactone	5-propyloxolan-2-one	105-21-5
	γ-Octalactone	5-butyloxolan-2-one	104-50-7
	γ-Undecalactone	5-heptyloxolan-2-one	104-67-6
Lactones#2	δ-Octanolactone	6-propyloxan-2-one	698-76-0
	δ-Nonalactone	6-Butyloxan-2-one	3301-94-8
	δ-Decalactone	6-pentyloxan-2-one	705-86-2
	δ-Dodecalactone	6-heptyloxan-2-one	713-95-1
	δ-Tetradecalactone	6-nonyloxan-2-one	2721-22-4
Sulfurs	dimethyl disulfide	(methyldisulfanyl)methane	624-92-0
	dimethyl trisulfide	(methyltrisulfanyl)methane	3658-80-8
	2,5-dimethylthiophene	2,5-dimethylthiophene	638-02-8
Terpenes	γ-terpinene	4-methyl-1-(1-methylethyl)-1,4-cyclohexadiene	99-85-4
	α-terpinene	4-methyl-1-(1-methylethyl)-1,3-cyclohexadiene	99-86-5
	citral	3,7-dimethylocta-2,6-dienal	5392-40-5
	linalool	3,7-Dimethylocta-1,6-dien-3-ol	78-70-6
	L(-)-Carvone	2-methyl-5-(prop-1-en-2-yl)cyclohex-2-en-1-one	6485-40-1
	geranyl acetate	3,7-dimethyl-2,6-octadien-1-yl acetate	105-87-3
Thiazoles	thiazole	1,3-thiazole	288-47-1
	benzothiazole	1,3-benzothiazole	95-16-9
	4,5-dimethylthiazole	4,5-dimethyl-1,3-thiazole	3581-91-7
Others	DEET	N,*N*-Diethyl-3-methylbenzamide	134-62-3
	Diethyl ether	Ethoxyethane	60-29-7
	Paraffin oil	x	8012-95-1

Supplementary table S3. Odor response profile (spikes/s) of nine T2 olfactory sensilla across the labella of female *An. coluzzii* to a panel of 20 odorant blends of 11 distinct chemical classes.

Blends	A - Cell								B - Cell							
	Zone-1		Zone-2		Zone-3		Zone-4		Zone-1		Zone-2		Zone-3		Zone-4	
	Spikes/sec	SE	Spikes/sec	SE	Spikes/sec	SE	Spikes/sec	SE	Spikes/sec	SE	Spikes/sec	SE	Spikes/sec	SE	Spikes/sec	SE
Alcohols#1	3.86	2.60	18.50	6.07	15.00	4.88	28.86	13.12	0.43	0.56	2.00	1.53	4.29	2.11	6.29	3.76
Alcohols#2	6.43	2.53	24.50	9.55	18.43	6.54	28.14	13.25	2.29	2.15	2.00	3.00	5.00	2.00	6.29	4.88
Alcohols#3	3.14	3.26	33.75	9.03	29.86	6.66	48.29	18.90	1.14	1.57	1.00	3.00	6.14	1.89	3.43	7.09
Aldehydes	43.86	19.14	66.00	9.18	49.00	12.19	64.86	21.07	5.71	4.12	1.00	1.15	4.14	2.53	4.29	5.29
Amines#1	4.00	3.96	1.50	2.74	-0.71	2.88	1.86	4.42	1.57	1.91	-1.25	2.26	-0.43	1.41	2.29	2.60
Amines#2	0.86	1.21	0.75	1.57	2.29	2.30	3.86	4.03	1.29	1.81	1.00	1.29	0.86	0.86	1.57	2.41
Amines#3	3.71	3.31	9.50	7.60	18.29	2.76	34.71	10.74	2.29	2.38	2.75	1.21	4.71	1.62	11.57	8.06
Acids#1	42.86	20.65	30.50	16.90	9.86	5.15	5.43	3.11	4.29	5.40	6.50	3.39	2.71	3.28	5.43	5.29
Acids#2	26.43	14.24	34.75	18.29	18.14	4.57	22.71	8.86	7.29	3.33	6.50	3.29	4.14	1.85	7.00	3.42
Acids#3	0.71	0.64	-0.50	2.35	1.57	2.04	1.14	1.38	-0.71	0.89	0.25	0.35	0.86	0.86	1.57	0.99
Esters#1	1.00	0.91	5.50	6.89	4.29	4.39	8.00	4.26	-0.86	1.04	4.75	5.40	2.14	1.19	4.14	2.53
Esters#2	0.57	1.82	5.25	5.58	17.00	7.42	32.86	12.53	-0.29	2.88	3.25	1.21	4.14	2.18	4.00	2.74
Indoles	0.57	1.14	19.75	11.39	19.14	5.85	28.29	14.29	-0.14	1.04	3.25	3.23	3.57	2.41	3.57	2.94
Ketones#1	15.86	5.30	30.25	7.78	24.43	7.93	41.43	16.06	3.43	2.37	3.75	2.34	5.29	3.15	4.86	4.48
Ketones#2	32.43	12.09	28.50	2.97	9.00	4.53	26.86	11.18	5.57	3.04	4.50	3.39	1.71	2.44	6.14	4.03
Lactones#1	5.00	3.97	6.25	3.85	10.43	5.00	10.86	6.28	2.14	1.75	3.75	2.73	0.57	2.19	6.43	4.76
Lactones#2	1.57	1.07	3.75	2.11	1.57	1.77	3.86	2.90	0.71	0.91	2.50	3.58	0.29	0.53	4.29	3.91
Sulphurs	0.71	0.89	1.50	0.91	3.14	2.40	8.43	4.72	0.43	1.07	1.75	0.89	1.14	1.19	3.71	3.60
Terpenes	66.71	21.61	2.00	1.53	1.57	3.39	1.14	3.55	6.00	2.81	0.75	0.68	-0.43	1.73	2.00	3.39
Thiozoles	37.71	14.41	48.50	9.35	39.00	13.55	58.00	14.67	5.14	3.82	4.25	1.86	5.57	3.41	6.71	2.30
Paraffin oil	-0.29	0.89	0.50	0.68	0.29	1.57	2.00	1.68	0.43	0.80	1.25	0.00	-0.29	1.35	0.86	1.55
Diethyl ether	-0.86	0.53	-0.75	0.91	1.29	2.19	1.00	3.11	-0.14	0.56	0.00	0.89	-0.57	1.21	1.86	2.46

Supplementary table S4. Odor response profile (spikes/s) of four T2 olfactory sensilla across the labella of female *An. coluzzii* to a panel of 81 unitary odorants.

Class	Compounds	A - Cell							B - Cell						
		Zone-1		Zone-2*	Zone-3		Zone-4		Zone-1		Zone-2	Zone-3		Zone-4	
		Spikes/sec	SE	Spikes/sec	Spikes/sec	SE	Spikes/sec	SE	Spikes/sec	SE	Spikes/sec	Spikes/sec	SE	Spikes/sec	SE
Alcohols	1-pentanol	5.0	0.5	2.0	1.0	1.0	9.5	2.5	4.0	3.5	3.0	1.0	3.0	1.5	0.5
	3-hexanol	3.0	1.5	3.5	-1.0	0.0	3.0	1.0	-0.5	0.5	1.0	1.5	1.5	2.0	3.0
	1-heptanol	2.0	2.5	3.0	2.5	0.5	2.5	0.5	1.5	2.0	1.5	2.0	0.0	0.0	1.0
	3-Octanol	2.0	3.5	10.5	6.0	2.0	19.0	2.0	2.5	1.5	3.0	1.5	2.5	3.5	0.5
	1-Octanol	9.0	1.5	7.5	3.5	0.5	5.0	1.0	0.0	1.0	1.0	2.5	1.5	0.5	0.5
	1-Octen-3-ol	9.0	3.5	16.0	4.5	0.5	2.0	1.0	0.0	0.5	2.0	3.0	1.0	1.5	0.5
	2-Nonanol	3.0	0.5	1.5	0.5	0.5	19.5	4.5	0.5	0.0	0.5	0.5	0.5	2.5	3.5
	1-undecanol	-2.0	3.0	3.5	0.5	0.5	0.5	0.5	2.0	1.5	2.5	1.0	1.0	1.0	1.0
	3-methyl-2-cyclohexen-1-ol	5.0	4.0	15.5	11.5	0.5	6.0	2.0	4.5	2.5	4.5	0.5	0.5	0.5	0.5
	2-methyl-3-buten-2-ol	8.0	0.5	8.0	0.5	0.5	2.5	0.5	1.5	0.5	1.5	0.5	0.5	0.0	0.0
	1-hexen-3-ol	3.0	1.5	1.5	1.5	1.5	8.5	4.5	-1.0	1.5	-1.0	1.0	1.0	2.0	1.0
	cis-3-hexen-1-ol	-1.0	2.0	3.5	1.0	1.0	3.5	1.5	2.0	0.0	1.5	0.0	0.0	-1.0	1.0
	trans-2-hexen-1-ol	2.0	0.0	4.0	2.5	0.5	0.5	0.5	3.5	2.0	3.5	-0.5	0.5	0.5	0.5
Aldehydes	Decanal	0.5	1.5	2.0	5.0	1.0	-0.5	0.5	1.5	1.5	-2.0	0.5	0.5	1.0	0.0
	Heptanal	3.5	1.5	1.0	1.0	1.0	2.0	1.0	1.0	2.0	-1.0	1.0	2.0	-0.5	0.5
	Octanal	0.5	0.5	6.0	0.5	1.5	0.5	1.5	1.0	0.0	0.0	2.0	1.0	1.5	0.5
	Hexanal	5.0	3.0	2.0	1.5	0.5	5.5	2.5	2.5	0.5	-1.0	1.0	1.0	1.5	0.5
	Phenylacetaldehyde	1.0	1.0	1.0	0.0	2.0	1.5	1.5	0.5	0.5	2.0	1.5	1.5	2.0	2.0
	Benzaldehyde	43.5	8.5	52.0	24.0	2.0	39.0	9.0	-0.5	0.5	-1.0	3.5	1.5	5.0	4.0
	trans-2-hexenal	5.0	3.0	-2.0	2.0	1.0	2.5	1.5	0.0	1.0	2.0	-0.5	1.5	2.5	2.5
Acids	Pyruvic acid	0.5	1.5	6.0	0.5	0.5	3.0	2.0	1.0	1.0	0.0	2.0	0.0	1.0	1.0
	L-(+)-Lactic acid	3.5	1.5	-3.0	-3.0	1.0	1.0	1.0	0.5	0.5	0.0	0.0	0.0	0.5	0.5
	Butyric acid	8.0	1.0	5.0	1.5	1.5	0.5	0.5	7.5	2.5	-2.0	0.0	0.0	0.0	0.0
	2-oxobutyric acid	6.0	3.0	3.0	0.5	1.5	0.5	0.5	0.5	1.5	0.0	-1.5	1.5	1.0	0.0
	isobutyric acid	1.5	0.5	7.0	0.0	0.0	0.5	2.5	0.5	0.5	-1.0	0.0	0.0	-0.5	0.5
	Valeric acid	41.0	4.0	15.0	-2.5	0.5	2.5	1.5	1.5	3.5	-1.0	0.0	0.0	1.0	0.0
	Isovaleric acid	5.0	3.0	5.0	-1.0	1.0	3.5	0.5	3.5	1.5	2.0	-0.5	0.5	-0.5	1.5
	2-Oxovaleric acid	56.5	5.5	2.0	1.0	2.0	3.0	3.0	2.5	1.5	1.0	0.5	0.5	3.5	3.5
	3-methylpentanoic acid	2.5	1.5	7.0	-1.0	0.0	1.5	1.5	1.5	1.5	-2.0	-0.5	1.5	0.0	1.0
	2-methylhexanoic acid	1.5	1.5	20.0	4.5	5.5	7.5	1.5	1.5	0.5	-2.0	1.0	1.0	0.0	0.0
	trans-2-methyl-2-pentenoic acid	25.5	2.5	26.0	-0.5	0.5	25.0	4.0	4.0	2.0	0.0	2.0	2.0	1.5	0.5
	Hexanoic acid	40.0	7.0	5.0	-0.5	0.5	4.0	2.0	1.5	0.5	1.0	3.0	0.0	-0.5	1.5
	Heptanoic acid	7.5	4.5	11.0	3.5	1.5	7.5	3.5	0.5	0.5	0.0	1.5	0.5	1.5	0.5
	octanoic acid	2.0	2.0	4.0	2.5	0.5	1.0	2.0	0.5	0.5	1.0	-1.0	0.0	0.5	0.5
	2-methylheptanoic	18.0	3.0	20.0	7.5	6.5	3.0	1.0	3.0	1.0	2.0	3.0	0.0	2.0	1.0
	Phenylacetic acid	1.5	0.5	2.0	4.0	4.0	4.5	0.5	0.5	1.5	0.0	-0.5	0.5	-2.0	1.0
	nonanoic acid	4.0	1.0	5.0	1.0	0.0	1.5	0.5	0.5	0.5	-1.0	0.0	0.0	-0.5	0.5
	oleic acid	2.5	0.5	3.0	0.0	3.0	3.0	1.0	-0.5	1.5	1.0	0.5	0.5	0.5	0.5
	undecanoic acid	1.5	0.5	4.0	0.5	0.5	-0.5	0.5	0.5	0.5	1.0	1.5	0.5	0.5	0.5
	lauric acid	-0.5	1.5	-2.0	1.5	1.5	0.5	0.5	-0.5	0.5	0.0	0.0	2.0	3.0	2.0
	tridecanoic acid	3.0	3.0	-1.0	0.0	0.0	1.5	0.5	0.0	1.0	0.0	-0.5	2.5	1.0	1.0
Esters	Isobutyl acetate	1.5	0.5	2.0	-1.0	4.0	3.5	1.5	1.0	0.0	-1.0	1.5	0.5	1.0	0.0
	Isoamyl acetate	1.5	2.5	1.0	1.0	1.0	2.5	0.5	2.5	0.5	0.0	2.0	0.0	0.5	0.5
	Phenylethyl acetate	0.5	0.5	9.0	12.0	7.5	14.5	2.5	2.0	2.0	0.0	2.5	0.5	0.0	0.0
	Methyl salicylate	1.5	0.5	2.0	3.0	1.0	10.0	1.0	1.0	0.0	1.0	1.0	0.0	0.0	0.0
	Dodecyl acetate	-0.5	0.5	-1.0	1.0	0.5	7.0	2.0	4.0	2.0	1.0	-1.0	1.0	-1.0	2.0
	Ethyl formate	1.0	4.0	8.0	-1.0	3.5	2.0	1.0	0.0	2.0	0.0	2.5	0.5	0.5	0.5
	ethyl propionate	1.5	0.5	4.0	-1.0	3.0	1.5	0.5	0.0	1.0	1.0	1.0	1.0	0.0	0.0
	Benzyl salicylate	1.5	0.5	3.0	0.0	1.0	1.0	0.0	1.0	1.0	1.0	0.0	0.0	-0.5	1.5
	Benzyl benzoate	1.5	0.5	3.0	5.0	1.0	0.0	2.0	0.0	1.0	2.0	2.0	0.0	-2.0	2.0
Indole	Indole	3.5	1.5	13.0	53.0	6.0	31.0	7.0	0.5	0.5	1.0	3.5	3.5	1.0	2.0
	skatole	7.0	1.0	19.0	19.0	7.0	20.0	4.0	0.5	1.5	-2.0	1.0	1.0	1.0	0.0
Ketones	3-Pentanone	6.0	3.0	3.0	2.5	0.5	2.0	1.0	4.5	2.5	0.0	0.5	0.5	1.5	0.5
	Cyclohexanone	12.5	1.5	2.0	3.0	1.0	3.0	1.0	2.5	2.0	2.0	0.5	0.5	0.0	0.0
	3-octanone	10.5	1.5	4.0	0.5	0.5	2.5	0.5	4.0	1.0	0.0	-0.5	0.5	0.5	0.5
	Sulcatone	14.0	1.0	8.0	1.0	1.0	1.5	0.5	0.5	1.5	1.0	-0.5	0.5	1.5	0.5
	2'-hydroxyacetophenone	17.5	1.5	12.0	0.0	0.0	18.5	1.5	5.0	1.0	1.0	1.0	1.0	4.0	2.0
	2-nonanone	7.5	1.5	2.0	3.5	2.5	1.5	1.5	4.0	2.0	-2.0	0.5	0.5	-1.0	2.0
	L(-)-carvone	7.0	1.0	1.0	0.5	0.5	14.0	2.0	0.5	0.5	0.0	0.5	0.5	1.0	0.0
	(+)-fenchone	10.0	3.0	0.0	1.5	0.5	1.0	1.0	1.5	3.5	2.0	0.0	0.0	1.0	1.0
	2',4'-Dimethylacetophenone	57.5	4.5	20.0	8.5	4.5	15.0	9.0	10.0	1.0	3.0	0.5	0.5	1.5	2.5
	acetophenone	40.0	8.0	19.0	16.0	4.0	17.0	4.0	2.0	1.0	0.0	1.0	1.0	3.0	1.0
	2-undecanone	5.5	1.5	2.0	0.0	0.0	-0.5	0.5	3.5	1.5	1.0	0.5	0.5	1.0	0.0
Terpenes	gamma-terpinene	10.0	9.0	5.0	5.0	3.0	1.5	0.5	0.5	0.7	0.0	1.5	1.5	1.0	1.0
	Alpha-terpinene	4.5	1.5	2.0	3.5	2.5	1.5	2.5	3.0	2.8	-1.0	2.0	1.0	1.5	0.5
	Citral	52.0	10.0	8.0	6.0	3.0	4.5	1.5	8.0	11.3	3.0	3.5	1.5	1.5	0.5
	Linalool	5.5	1.5	13.0	10.0	1.5	2.5	1.5	2.0	4.2	1.0	2.5	1.5	0.5	0.5
	Geranyl acetate	17.0	1.0	4.0	4.5	3.5	2.0	2.0	5.0	5.7	2.0	2.0	1.0	1.5	1.5
Thiozoles	Thiozole	15.5	3.5	15.0	4.5	1.5	2.5	0.5	1.5	3.5	1.0	1.5	1.5	-0.5	0.5
	benzothiazole	36.0	2.0	24.0	37.5	5.5	23.0	6.0	7.0	5.7	2.0	0.5	0.5	1.5	1.5
	4,5-dimethylthiazole	28.5	3.5	20.0	7.5	1.5	7.0	5.0	3.0	1.4	0.0	2.5	1.5	0.5	0.5
Amines	Butylamine	2.0	1.0	4.0	-1.0	1.0	2.0	1.0	1.5	0.5	0.0	1.5	0.5	0.5	0.5
	Pentylamine	3.0	1.0	5.0	1.0	0.0	4.5	3.5	0.5	0.5	2.0	0.0	0.0	3.0	1.0
	Heptylamine	-0.5	1.5	2.0	0.5	0.5	7.0	1.0	-2.0	1.0	-1.0	0.5	0.5	0.5	0.5
	Pyrrolidine	3.5	0.5	2.0	2.0	1.0	2.0	2.0	0.5	0.5	0.0	0.5	0.5	0.5	0.5
	Cadaverine	1.0	1.0	-1.0	0.0	1.0	1.0	1.0	1.5	0.5	1.0	2.0	2.0	-0.5	0.5
	Pyrrole	-0.5	0.5	4.0	1.0	0.0	11.5	3.5	-0.5	0.5	1.0	0.5	0.5	-1.0	2.0
	2-methylpyrazine	8.5	1.5	11.0	6.5	0.5	1.0	1.0	1.5	0.5	-1.0	1.0	1.0	0.5	0.5
	Triethylamine	0.5	1.5	2.0	0.5	0.5	4.5	1.5	0.0	0.0	2.0	2.0	1.0	3.0	1.0
	Ethylpyrazine	0.5	0.5	4.0	12.0	4.0	18.5	8.5	0.0	1.0	1.0	3.0	1.0	-0.5	0.5
Others	DEET	11.5	3.5	2.0	-1.5	2.0	4.0	2.0	-1.0	3.0	1.0	0.0	0.5	1.0	1.0
	diethyl ether	1.5	1.0	0.5	2.0	1.5	3.0	1.0	2.0	0.5	1.0	1.0	1.5	1.0	0.5
	paraffin oil	0.5	1.5	1.0	0.5	0.9	1.0	0.5	1.0	0.5	0.6	1.0	0.5	1.0	1.5

Supplementary table S5. List of *An. coluzzii* labella (SSR) active 10-component odorant blend

Compounds	Spikes/s
Benzaldehyde	52
trans-2-methyl-2-pentanoic acid	26
Hexanoic acid	40
Valeric acid	41
2-oxovaleric acid	57
Indole	31
Acetophenone	58
2,4-dimethylacetophenone	40
4,5-dimethylthiazole	29
Benzothiazole	43
